# Complete Third Nerve Palsy With Pupil Involvement in Herpes Zoster Ophthalmicus: A Case Report

**DOI:** 10.7759/cureus.76672

**Published:** 2024-12-31

**Authors:** Nur Syahirah Abd Karim, Azhany Yaakub, Tengku Norina Tuan Jaffar, Rafikah Mahadi

**Affiliations:** 1 Department of Ophthalmology, Universiti Sains Malaysia School of Medical Sciences, Kota Bharu, MYS; 2 Department of Ophthalmology, Raja Perempuan Zainab Il Hospital, Kota Bharu, MYS; 3 Department of Ophthalmology, Universiti Kebangsaan Malaysia Medical Centre, Kuala Lumpur, MYS

**Keywords:** anisocoria, cranial nerve palsy, herpes zoster ophthalmicus, hzo, ophthalmoplegia

## Abstract

Simultaneous complete oculomotor nerve palsy in herpes zoster ophthalmicus (HZO) is rare. We report a case of a 65-year-old lady who presented with a right drooping eyelid for 2 days associated with rashes over her right forehead. Examination showed crusted vesicular lesions on the right V1 dermatome with right complete ptosis, anisocoria, and limited right extraocular movement on all gazes except full movement on abduction. The anterior chamber showed mild right eye anterior chamber inflammation and bilateral fundus examinations were normal. Other neurological examinations were normal. Neuroimaging also was normal. A diagnosis of complete third nerve palsy secondary to HZO with keratouveitis was made. HZO-causing cranial nerve palsy is a relatively uncommon finding. In severe cases with significant nerve involvement, additional treatments or interventions may be necessary to manage the third nerve palsy and its associated complications.

## Introduction

Herpes zoster ophthalmicus (HZO) is a reactivation of the childhood chicken pox virus (varicella zoster) along the ophthalmic division of the fifth cranial nerve (CN V1) which often manifests as a vesicular rash or dermatitis [1]. Infection with herpes zoster, also known as shingles, is caused by varicella zoster virus (VZV) in individuals who have already been exposed to the virus, either through vaccination or wild type [2]. HZO occurs as a result of reactivation in the trigeminal ganglion. Conjunctivitis, keratitis, uveitis, and secondary glaucoma are the common findings associated with HZO. Cranial nerve palsies and optic nerve involvement rarely occur either as sequelae or early in the course of the disease [3]. The following case report discusses a patient who developed a complete third nerve palsy with optic nerve involvement after being diagnosed with HZO.

## Case presentation

A 65-year-old lady with underlying diabetes mellitus and hypertension presented with a right drooping eyelid, right eye reduced vision, and a vesicular skin lesion over the right forehead for three days.

**Figure 1:**
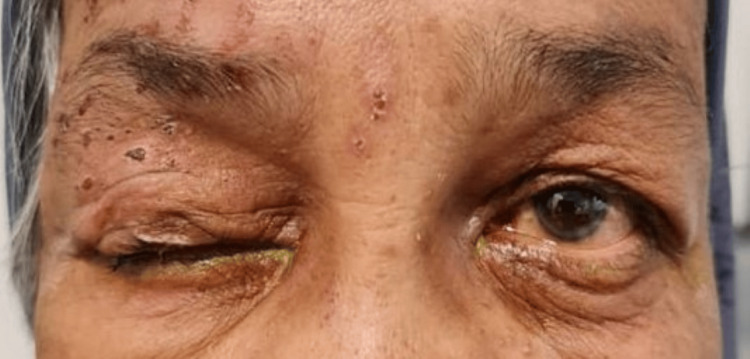
Vesicular lesion over the right side of the forehead with herpes zoster-induced complete ptosis over the right eye.

**Figure 2:**
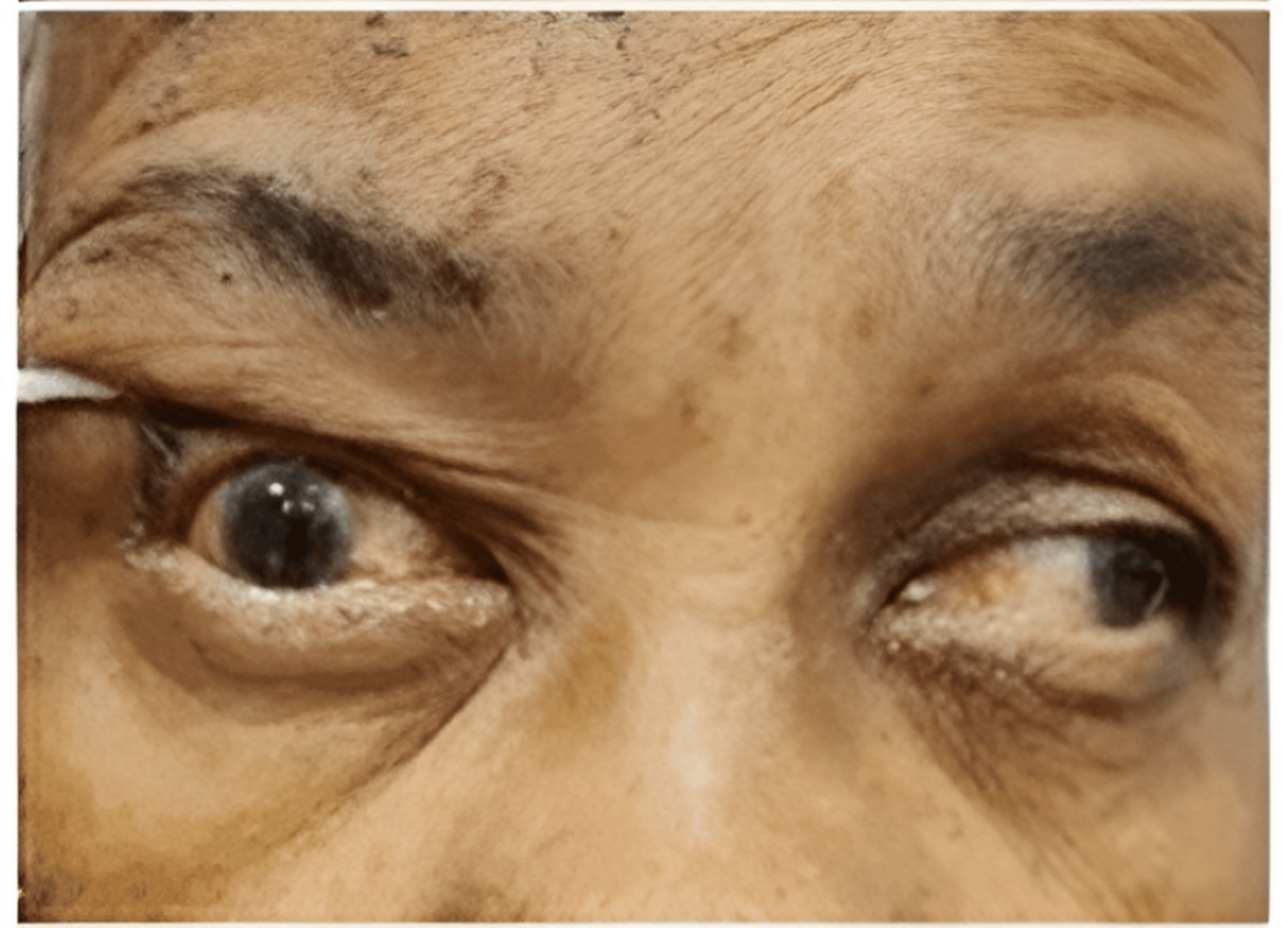
Limitation of adduction over the right eye.

**Figure 3:**
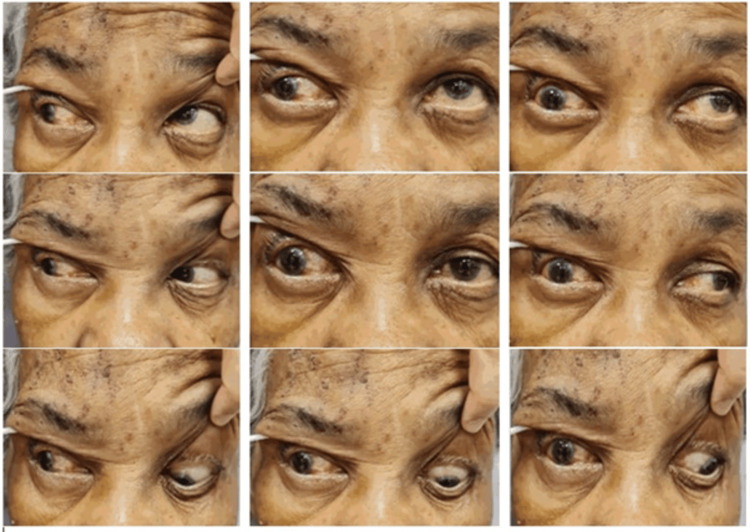
Nine cardinal gazes demonstrating limited extraocular muscle movement except for abduction over the right eye.

**Figure 4:**
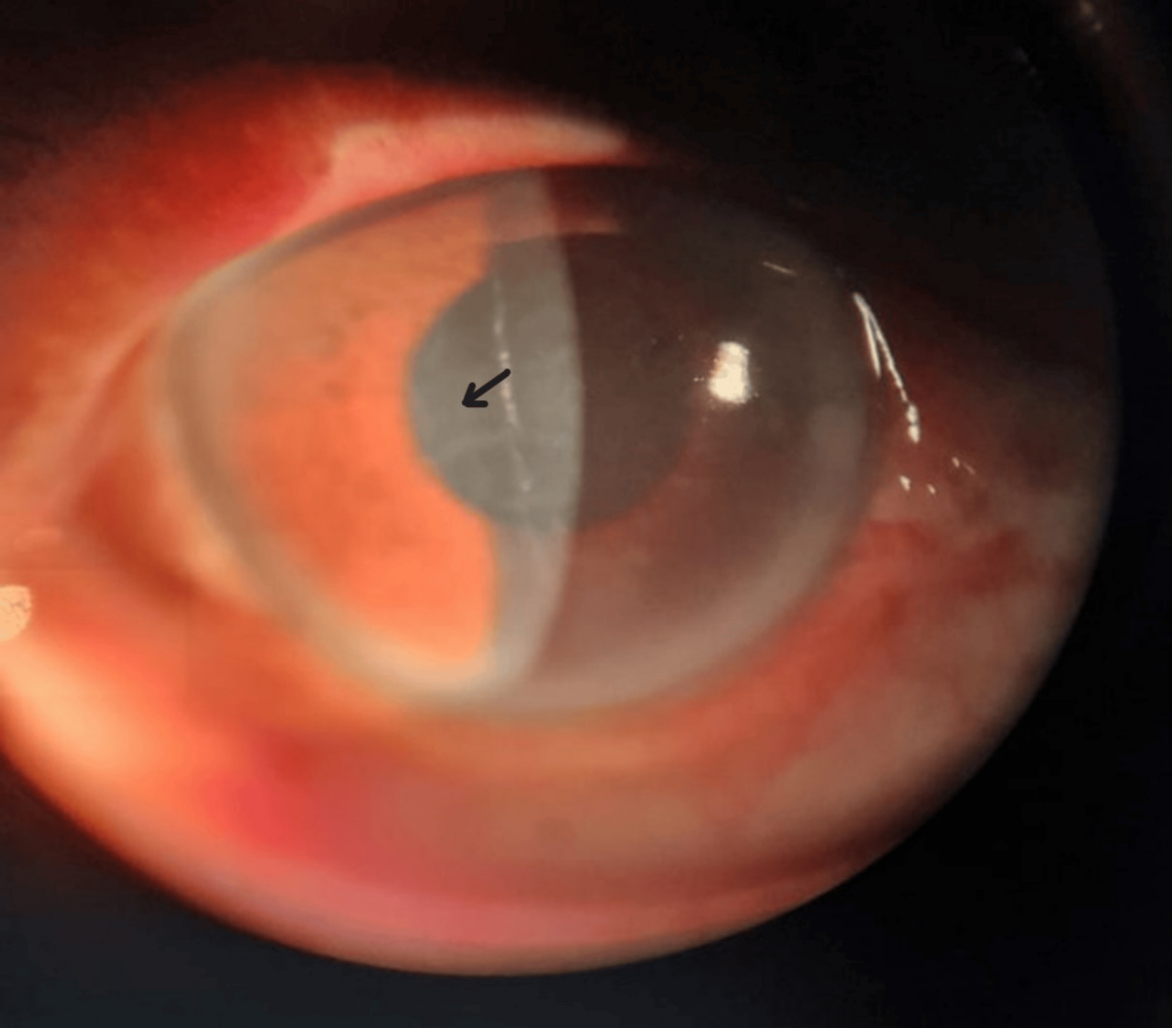
The slit-lamp examination showed keratouveitis. Arrow shows endothelial straie with anterior chamber cells 2+.

On examinations, the patient had a vesicular lesion over the right side of the forehead belonging to the V1 dermatome (Figure 1). There was a complete ptosis over the right upper lid. Her visual acuity over her right eye was hand movement and her left eye was 6/36 with pinhole 6/12. During extraocular motility testing, restriction over all gazes except abduction for the right eye was observed (Figures 2-3). She had anisocoria in which the right eye was mid-dilated 4 mm and reactive pupil and the left eye 2 mm pupil. The relative afferent pupillary defect was absent. A slit-lamp examination revealed a mildly injected conjunctiva. The cornea sensation was reduced with the presence of endothelial striae. However, there was no epithelial defect or corneal lesion. There was the presence of cell 2+ in the anterior chamber (Figure 4) but no hypopyon and posterior synechiae. Intraocular pressures for both eyes were normal. Fundus examination showed a normal disc and macula. The left eye was normal.

Other cranial nerve functions and systemic examinations were normal. Her white cell counts, as well as blood sugar level and blood pressure, were within normal limits (Table 1).

**Table 1:** Blood investigations

	Result	Normal Range
Full Blood Count		
Total white cell	6.38 × 10^9/L	4.0-10.0 × 10^9/L
Hemoglobin	125 g/L	120-150 g/L
Platelet	393 × 10^9/L	150-410 × 10^9/L
Renal Profile		
Creatinine	46 umol/L	45-84 umol/L
Urea	2.9 mmol/L	2.8-7.2 mmol/L
Sodium	134 mmol/L	136-146 mmol/L
Potassium	3.5 mmol/L	3.5-5.1 mmol/L
HbAIC	7.7 %	>6.3 % type 2 diabetes mellitus

She was diagnosed with right herpes zoster ophthalmicus with keratouveitis and right third nerve palsy with pupillary involvement.

Computed tomography (CT) of the brain was performed to exclude other causes of third cranial nerve palsy as well as life-threatening causes. No space-occupying lesion and posterior communicating artery aneurysm were found.

A treatment course consisting of oral acyclovir 800 mg five times daily for 14 days, ointment acyclovir 3% five times daily, and topical gentamicin sulfate 0.3% with betamethasone 0.1% four times daily. Oral acyclovir 800 mg five times daily was tapered to 400 mg BD for another 14 days as a prophylactic dose.

## Discussion

Fifty percent of HZO cases may result in ocular complications, which typically fall into one of four categories: keratitis, iritis, muscle palsies, and optic neuritis [1]. It is uncommon for the cranial nerve that controls extraocular muscle to be involved in the course of HZO, which is attributed to 1.1%- 2.9% of the incidence rate [3]. 

The most commonly involved was the oculomotor nerve, followed by the abducens nerve and trochlear nerve [4]. Adduction and vertical ocular motility deficits are caused by cranial nerve III palsy when the nerve is affected, and the pupil can be affected as well because of the course of the pupillary fibers through cranial nerve III [1].

Complete third nerve palsy can be caused by various causes and can include space-occupying lesions, aneurysmal compression, microvascular complications, inflammation, infections, and trauma. Oculomotor nerve palsy, which can manifest as diplopia, mydriasis, and ptosis, appears to be the most frequent cause of ophthalmoplegia. Ptosis results from a disruption in the levator palpebrae superioris muscle supply and diplopia results from a breakdown in the extraocular muscles supplied by the cranial nerve. Whereas mydriasis develops when the parasympathetic fibers that run along the nerve are affected [5]. In our case, the patient showed anisocoria, ptosis, restricted in all gazes except abduction, which is suggestive of complete third nerve palsy.

Despite the typical history of HZO, this patient had complete right third nerve palsy with pupil involvement which necessitated the neuroimaging.

In order to rule out a slowly compressive lesion or an aneurysm, immediate central nervous system imaging is advised for all third nerve palsies, regardless of whether the pupils are involved or spared. A patient with total third nerve palsy and complete pupil sparing is one potential exception [3].

Seventy-five percent of cases will have HZO-induced ophthalmoplegia with a range of 2 to 42 days after the onset of herpes zoster rashes. The average of cases occurs 9.5 days after the onset. Despite not being the typical manifestation, ophthalmoplegia has been observed to occur concurrently with the HZO onset [1]. However, our patient had acute ophthalmoplegia and ptosis within the first day of presentation.

In most cases, the ophthalmoplegia is self-limiting and resolves entirely or almost entirely on its own in 4.4 months on average in 65-76.5% of those who are affected [1]. The duration varies and can range from 2 weeks to 1.5 years. However, restricted ocular mobility and/or persistent ptosis in certain patients may persist continuously [3]. In our case, the ptosis and ophthalmoplegia had not improved despite two months after the onset.

Systemic antiviral medications (acyclovir, valacyclovir, or famciclovir) should be taken within 72 hours of the development of rashes to improve dermatologic healing, decrease pain, and lower the incidence of HZ and HZO sequelae [6]. Our patient received oral acyclovir 800 mg five times daily for two weeks and was continued with 400 mg for another two weeks as a prophylactic dose. The last follow-up was two months before this report was written. No recurrence was detected.

Two months after treatment, her right eye visual acuity improved to 6/60. Her right ocular motility had a slightly improved restriction on adduction, elevation, and suppression. Her cornea had resolved keratouveitis. However, there was still complete ptosis and a dilated pupil of 4 mm.

There are several reported cases of HZO with complete third nerve palsy with pupil involvement (Table 2). Most of the cases had a slight improvement in ophthalmoplegia after 2 weeks of presentation.

**Table 2:** Summary of reported cases of herpes zoster ophthalmicus with complete third nerve palsy

	Low et al. [6]	Harthan and Borgman [1]	Delengocky and Bui [7]	Hakim et al. [8]	Our Case
Age/Sex	78/F	84/F	72/M	87/F	65/F
Time from rash to onset of ophthalmoplegia (CN3) (day)	5	7	42	6	3
Ptosis	Complete Ptosis	Complete Ptosis	Complete ptosis	Complete ptosis	Complete ptosis
Anisocoria	-	+	+	+	+
Other Ocular Manifestation					
Keratitis/Keratouveitis	Keratouveitis	Keratitis	-	-	Keratouveitis
Other cranial nerve palsy CN 4 CN 6	+ -	- -	- -	- -	- -
Treatment	Oral Acyclovir 800 mg 5× per day + oral prednisolone 1 mg/kg/day for 6 weeks	Oral acyclovir 800 mg 5× per day for 7 days	Oral valacyclovir 1 g BD	Oral and topical acyclovir	Oral acyclovir 800 mg 5× per day + ointment acyclovir for 14 days
Recovery	1 year	6 months	Defaulted	2 weeks	

## Conclusions

When cranial nerve palsy is identified, prompt referral, imaging, and blood tests are crucial to rule out any potentially life-threatening. In our case, CT ruled out a posterior communicating artery aneurysm and/or space-occupying lesion. Once these conditions were ruled out, then we could confirm that the cranial palsy and pupil involvement were secondary to HZO. This case demonstrates the relatively uncommon case of ophthalmoplegia with pupil involvement induced by HZO.
